# Insight into the protein degradation during the broad bean fermentation process

**DOI:** 10.1002/fsn3.2879

**Published:** 2022-04-11

**Authors:** Hongbin Lin, Binbin Zhou, Jianhua Zhao, Shiqi Liao, Jinlin Han, Jiaxing Fang, Ping Liu, Wenwu Ding, Zhenming Che, Min Xu

**Affiliations:** ^1^ 12598 School of Food and Bio‐Engineering Xihua University Chengdu China

**Keywords:** broad bean fermentation, protease, protein degradation, the simulation system

## Abstract

Broad bean fermentation is of vital importance in PixianDouban (PXDB) production, as well as a key process for microorganisms to degrade protein, which lays the foundation for the formation of PXDB flavor. In this study, two fungi and bacteria were screened, and their morphology, molecular biology, growth, and enzyme production characteristics were analyzed, and then they were applied to the broad bean fermentation simulation system. The protein, peptide, amino acid, amino nitrogen, and sodium dodecyl sulfate‐polyacrylamide gel electrophoresis (SDS‐PAGE) in the system were evaluated. The results showed that the four microorganisms were *Aspergillus oryzae*, *Aspergillus jensenii*, *Staphylococcus gallinarum*, and *Enterobacter hormaeche*. *Aspergillus oryzae* had the highest protease activity at pH 7.0, while the other three strains had better enzyme activity stability under neutral acidic conditions. And the total protein (F1 and F2 were 18.32 g/100 g, 19.15 g/100 g, respectively), peptides (11.79 ± 0.04 mg/g and 12.06 ± 0.04 mg/g), and amino acids (55.12 ± 2.78 mg/g and 54.11 ± 1.97 mg/g) of the fungus experimental groups (F) were higher than the bacterial experimental groups (B). In addition, the enzyme system produced by fungi exhibited a stronger ability for albumin (20 kDa) and glutenin (<30 kDa) deterioration in neutral conditions, while the bacterial enzyme system was more efficient in degrading albumin (<30 kDa) and glutenin (20–30 kDa) in acidic conditions, as indicated by SDS‐PAGE. These findings showed that both bacteria and fungi played an important role in the degradation of protein in different fermentation stages of broad bean fermentation.

**Practical applications:**

There is a lack of comprehensive understanding of the protein composition and protein degradation mechanism of broad beans in the fermentation stage of PXDB. This research work explored the differences in the degradation of PXDB fermented protein by different microorganisms, and provided a theoretical basis for optimizing the production of PXDB and improving the quality of PXDB.

## Graphical abstract

In this study, two fungi and bacteria were screened, and their morphology, molecular biology, growth, and enzyme production characteristics were analyzed, and then they were applied to the broad bean fermentation simulation system. The protein, peptide, amino acid, amino nitrogen, and sodium dodecyl sulfate‐polyacrylamide gel electrophoresis (SDS‐PAGE) in the system were evaluated. We discovered and showed that both bacteria and fungi played an important role in the degradation of protein in different fermentation stages of broad bean fermentation.

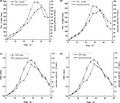

## 1 INTRODUCTION

**FIGURE 1:**
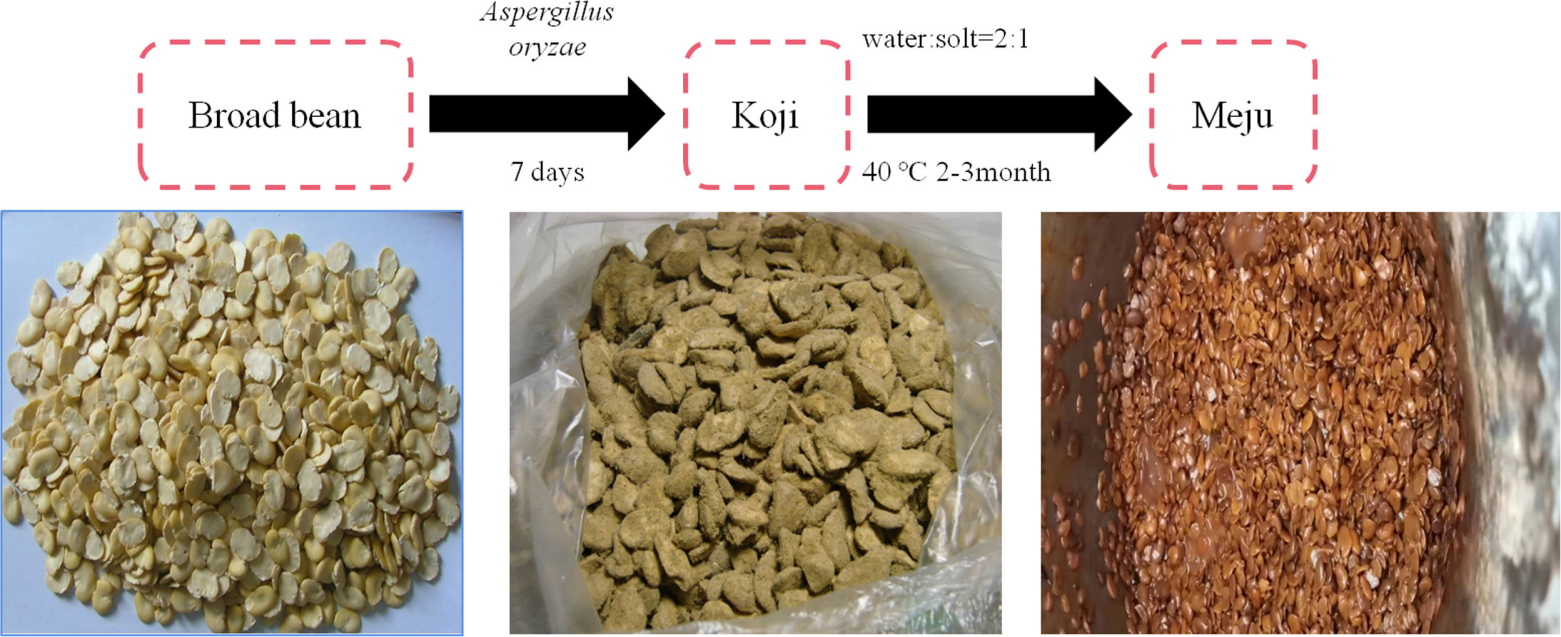
Broad bean Meju fermentation process

PixianDouban (PXDB) is a compound seasoning from Sichuan, China, with a history spanning several hundred years. It is regarded as the “the soul of Sichuan cuisine” due to its unique taste and flavor (Li, 2008). PXDB is produced using broad beans and peppers as the main raw materials and its production includes three predominant processes. First, the cut fresh peppers are mixed with brine and fermented at room temperature for 3 months. Then there is the broad beans fermentation stage in which shelled broad beans are mixed with wheat flour, inoculated with *Aspergillus oryzae* to produce Koji for 4–7 days, and then blended with brine and left at 40°C for 2–3 months to obtain Meju. Finally, the salted peppers and the Meju are mixed and fermented for at least 6 months to obtain a unique flavored PXDB. Particularly, broad bean fermentation is an essential process for PXDB production (Li et al., 2017). During the Meju stage (shown in Figure 1), the enzymes produced by the microorganisms break down the proteins into smaller molecules, such as polypeptides, free amino acids (FAAs) (Sanjukta & Rai, 2016), playing a vital role in PXDB flavor development. Researchers have shown that the accumulation of small peptides augments the sensory quality of PXDB during the fermentation process (Li et al., 2021).

The proteases produced by the microbes play an essential role during the fermentation process (Lin et al., 2016). As for PXDB fermentation stage, the accumulation and degradation of broad bean proteases lay the foundation for flavor substance formation during the next PXDB stage (Li et al., 2017). Proteases are primarily produced by microbial flora metabolism in the environment, denoting protein degradation. Previous studies have investigated that *Leuconostoc lactis*, *Staphylococcus xylosus*, *Staphylococcus succinus*, *Amylomyces rouxii*, *Mucor genevensis*, *Absidia corymbifera*, *Issatchenkia orientalis*, basidiomycete yeast sp., and *Metschnikowia pulcherrima* were only detected in traditionally fermented PXDB (Li et al., 2018). In addition, *Bacillus amyloliquefaciens*, *Bacillus methylotrophicus*, *Bacillus subtilis*, *Bacillus licheniformis*, and *A. oryzae* displayed a strong capacity for producing enzymes, such as α‐amylase, cellulase, xylanase, acid protease, and leucine aminopeptidase, which contributed significantly to PXDB fermentation (Lu et al., 2020). Moreover, the intensity of the corresponding enzymes in the bacterial community influenced the organic acid content, while both the bacterial and fungal enzymes impacted the FAAs and volatile compound characteristics (Yang et al., 2021). The water‐soluble fraction of traditional Doenjang was found to contain a group of taste compounds, such as salts, amino acids, organic acids, and peptides, produced during proteolysis (Kim & Lee, 2003). The other study has found that both *Lactobacillus plantarum* and *Saccharomyces cerevisiae* accelerated protein degradation in duck meat. *L. plantarum* has the capacity of alleviating lipid oxidation in cured duck leg meat, whereas *S. cerevisiae* tends to improve the meat taste better (Cai et al., 2020). However, minimal studies are available regarding the impact of microbial differences on protein degradation during broad bean fermentation.

Therefore, two fungi and two bacteria were screened during the broad bean fermentation stage, and their morphology and 16S RNA and internally transcribed spacer (ITS) sequencing were used to identify the growth curves and enzyme production curves of the four strains. The selected strains were applied to broad bean fermentation process and a simulation system was established to study the changes of total protein, main protein, peptide, amino acid, amino acid nitrogen, and SDS‐PAGE in each system to explore the protein degradation mechanism during the broad bean fermentation process.

## 2 MATERIALS AND METHODS

### 2.1 Sampling and preparation

The prefermentation PXDB samples were obtained from the Chengdu Wangfeng Food Co., Ltd. workshop. The samples consisted of four diagonal points and a center point at 1.5 m from the fermentation tank (Zhu et al., 2017) (each point was about 200 g). The experimental samples were transferred to a uniform sample bag immediately after mixing and stored in a refrigerator at −20°C until use.

### 2.2 Strain screening and identification

Here, 90 ml of physiological salt was added to a 10 g sample, shaken well, and left to stand. Next, 1 ml of the subsequent supernatant was diluted sequentially to obtain 10^–2^, 10^–3^, 10^–4^, and 10^–5^ times the bacterial suspension, after which 0.1 ml of each dilution was spread on a medium primary screening plate. The plates were incubated at 30°C for 24–36 h (Cheng et al., 2018). The strains with prominent hydrolysis circles were then selected for further streaking and separation, while those with a larger ratio of hydrolysis zone to colony diameter (D/d) than the primary screening strains were chosen (Merin et al., 2015).

The initially screened fungi and bacteria were inoculated into the basic fermentation medium and cultured via shaking at 30°C at 200 revolutions per min (rpm) and 37°C at 200 rpm for 48 h, respectively. The fermentation broth was centrifuged at 3000 rpm for 10 min, after which enzyme activity was measured using the Folin method (Mani et al., 2015). Then, the strains displaying high enzyme activity were screened.

The phenotypic and biochemical characteristics were determined according to Wang et al. (2018). The selected fungi and bacteria were inoculated into potato dextrose agar medium and plate count agar medium, respectively, and cultured at 28°C for 3 days and 37°C for 3 days.

Genomic DNA was extracted from the isolated single strain using a TIANamp DNA Kit (Beijing Tiangen Biochemical Co., Ltd., China) according to the manufacturer's instruction. The V3–V4 region of bacterial 16S rRNA genes and the ITS region of fungal ribosomal RNA (rRNA) genes were the polymerase chain reaction (PCR) amplified using two universal primer sets, 27 forward (F) (5′‐AGAGTTTGATCCTGGCTCAG‐3′)/1492 reverse (R) (5′‐GGTTACCTTGTTACGACTT‐3′) (Adhikari et al., 2020; Zhang et al., 2019) and internal transcribed spacer 1 (ITS1) (5′‐TCCGTAGGTGAACCT GCGG‐3′)/internal transcribed spacer 4 (ITS4) (5′‐TCCTCCGCTTATTGATATGC‐3′) (Kim, Koh, et al., 2017). The PCR amplification products were detected using agarose gel electrophoresis and sequenced by the Chengdu Qingke Biotechnology Company. The sequencing results were submitted to GenBank for similarity analysis. Finally, the phylogenetic tree was established using MEGA7.0 (Yu et al., 2021).

### 2.3 Analyzing the bacterial strain growth and enzymatic characteristics

The spore and bacterial suspensions of the four strains obtained via screening were prepared and inoculated into 50 ml of Luria‐Bertani medium at a ratio of 2% to obtain a seed medium. The samples were cultured via shaking at 37°C at 200 rpm. The supernatant was collected at 4 h intervals using a triangular flask to determine the enzyme activity. The OD (optical density) value of the bacterial culture solution was measured at 600 nm using the photoelectric turbidimetric method. Next, the mycelium of the fungal culture solution was collected, dried to a constant weight in an oven at 80°C, and weighed.

The seed solution of each strain was subjected to centrifugal freezing (8000 *g*, 4°C for 15 min) and passed through a 0.22‐µm filter membrane to obtain a crude enzyme solution. The impact of the pH values (4.0, 5.0, 6.0, 7.0, and 8.0) and NaCl concentrations (0%, 5%, 10%, 15%, and 20%) on the protease activity was determined according to a method described by Gao et al. (2010). using 1% casein as a substrate.

### 2.4 Preparing via the single‐strain Koji fermentation

After activating the culture twice, a 106 spores/ml spore suspension and a 106 CFU/ml bacterial suspension were prepared. The 1% preparation solution was inoculated into the seed warp medium and cultured for 72 h at 90% humidity and 30°C (Vilanova et al., 2000).

First, the broad beans were boiled in water for 1 min and then placed into cold water for 5 min. Next, 200 g of the broad bean mixture, 50 g of flour, and 0.5% of the strains were placed in a fermenter and cultured at 90% humidity and 30°C to obtain the broad bean Koji. The fermentation groups (FG) were numbered FG (F1), FG (F3), FG (B3), and FG (B5), respectively. Sampling occurred at 0, 3, and 7 days, and the specimens were labeled K0, K3, and K7, respectively. Broad bean Koji was mixed with 15% saltwater at a 1:1 ratio and placed in a fermenter after mixing evenly. The petals were cultured at 90% humidity and 37°C for 90 days while stirring and watering regularly. Sampling occurred at 15, 30, 45, 60, and 90 days during the horsebean Meju fermentation, and the specimens were labeled M 15, M 30, M 45, M 60, and M 90.

### 2.5 The impact of the different Koji fermentation strains on the degradation of pre fermentation protein

The total protein content in the samples during different prefermentation stages was determined using the automatic Kjeldahl method (Urbat et al., 2019).

The Osborne method was used to fractionate the protein according to the protein solubility differences. First, 5 g samples were weighed and extracted successively with distilled water, 5% NaCl, 75% ethanol, and 0.1 mol/L NaOH as extraction agents to obtain water‐soluble, salt‐soluble, alcohol‐soluble, and gluten solutions. The Bradford method was then employed to determine the primary protein content (Vondel et al., 2020).

To determine the polypeptide content (Krewing et al., 2020), 2 g of the samples was weighed and ground using a mortar, after which 0.1 mol/L Tris–HCl buffer solution (pH 8.8) was added until reaching 10 ml. The sample was extracted at 4°C for 1 h and centrifuged at 8000 rpm for 15 min to obtain the supernatant, which was mixed with 15% trichloroacetic acid at the same volume, and left to stand at 4°C for 30 min. The macromolecular proteins were removed by centrifugation at 8000 rpm for 15 min. The supernatant was considered the peptide extract, while the polypeptide content was determined using the Bradford method.

To determine the total FAAs (Khan et al., 2018), 2 g of the ground and pulverized samples was weighed and distilled to 50 ml, extracted at 4°C for 1 h, and centrifuged at 3500 rpm for 20 min to obtain the supernatant. Next, 1.0 ml each of the supernatant, sodium acetate (CH_3_COONa) buffer solution at pH 6.5, and a 2% ninhydrin solution were mixed to obtain the sample solution, which was boiled for 40 min and cooled for 15 min. A pure water solution was used as a blank control.

### 2.6 Establishing the fermentation simulation system

A 50 g of albumin and gluten powder was added to 0.05 mol/L phosphate buffer solution (pH 7.0) to prepare the protein culture medium at a concentration of 10 g/100 ml. A nonacidified simulation system (NA) was established to simulate the fermentation environment during the early stage of broad bean Meju fermentation. Here, 18 g of salt was added to 100 ml of albumin and gluten culture solution, respectively, while the acidified simulation system (A) was established by adding 18 g salt and 2 g Glucono Delta Lactone, respectively, to simulate the acidic environment during the late fermentation stage of broad bean Meju. The crude enzyme solution of each strain was inoculated into the albumin‐nonacidified simulation system and labeled as A‐NA (F1), A‐NA (F3), A‐NA (B3), and A‐NA (B5), respectively. Inoculation into the albumin‐acidified simulation system yielded A‐A (F1), A‐A (F3), A‐A (B3), and A‐A (B5), respectively. The control group without inoculation was labeled A‐C. Similarly, the crude enzyme solution of each strain was inoculated into the glutenin‐nonacidified simulation system and labeled G‐NA (F1), G‐NA (F3), G‐NA (B3), and G‐NA (B5), respectively. Inoculation into the glutenin‐acidified simulation system yielded G‐A (F1), G‐A (F3), G‐A (B3), and G‐A (B5), respectively. The control group without inoculation was labeled G‐C. Finally, all the simulated systems were incubated at 30°C and 200 rpm for 4 h.

### 2.7 The effect of the different strains on protein degradation in the simulated systems

The amino acid nitrogen content was determined via formaldehyde titration in the culture mediums of the albumin and gluten simulation systems (Zhao, Wei, et al., 2017).

The water‐soluble protein and glutenin simulation system culture solution was centrifuged at 8000 rpm at 4°C for 20 min to obtain the supernatant, after which SDS‐PAGE was performed. The extracted protein solutions were diluted with different proportions of buffer solution, after which the solution was prepared at a protein concentration of 0.1 mg/ml. Next, a 5X loading buffer solution was added (each mL containing 0.1 g SDS, 0.05 g bromophenol blue, 0.5 ml glycerol, 0.05 ml mercaptoethanol, and 0.25 ml 1 M pH 6.8 Tris–HCl) and boiled in water for 5 min. Extracellular proteins were resolved on 12% polyacrylamide gels, and the protein bands were visualized by staining with 0.1% Coomassie Brilliant Blue R‐250 (CBBR‐250) after electrophoresis (Zuo et al., 2018).

### 2.8 Statistical analysis

Each sample was replicated three times. All results were presented as means ± standard deviation (SD). Significant difference analysis was performed using SPSS 19.0. The statistical analyses were carried out using Origin 9.1 software.

## 3 RESULTS AND DISCUSSION

### 3.1 Strain screening and identification

Six fungal strains (F) and five bacterial strains (B) were screened via the formation of a visible transparent circle near the colony. As shown in Table 1, F1, F3, F4, and F6, and B1, B2, B3, and B5 with larger D/d values were selected for the next rescreening. Then eight strains were cultured using a shake flask. According to Table 2, the results showed that the protease activities of F1, F3, B3, and B5 were significantly higher than those of other strains, which were 192.45 ± 2.13, 163.15 ± 1.11, 123.59 ± 1.66, and 131.15 ± 0.47 U/ml, respectively. Therefore, these four strains were used as representative strains for the follow‐up study.

**TABLE 1:** Screening of the protease‐producing strains

Serial number	Diameter of the transparent ring D/mm	Diameter of the colony D/mm	D/d values
F1	16.2	7.2	2.25
F2	11.1	8.4	1.32
F3	15.3	7.1	2.15
F4	14.5	8.6	1.69
F5	14.3	6.9	1.62
F6	10.7	6.5	1.65
B1	13.2	6.5	2.03
B2	14.5	7.5	1.93
B3	15.7	6.1	2.57
B4	12.1	7.2	1.68
B5	13.5	5.9	2.29

Note

The six fungi are numbered F1–F6, and the five bacteria are numbered B1–B5.

**TABLE 2:** The protease activity of the protease‐producing strains

Serial number	Protease activity U/ml	Serial number	Protease activity U/ml
F1	192.45 ± 2.13	B1	76.75 ± 1.68
F3	163.15 ± 1.11	B2	89.12 ± 1.46
F4	98.15 ± 0.89	B3	123.59 ± 1.66
F6	101.11 ± 2.01	B5	131.15 ± 0.47

**FIGURE 2:**
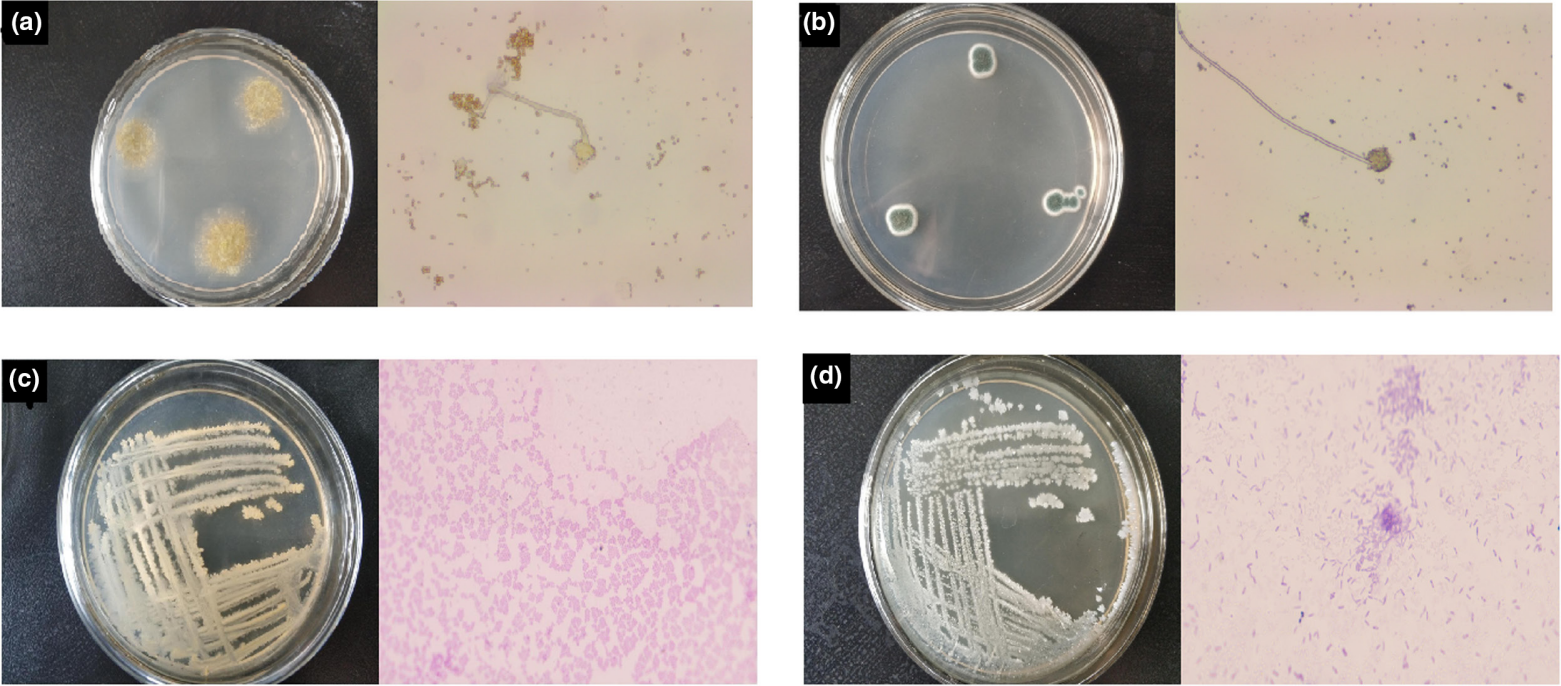
The colonial and individual strain morphology: strain F1 (a); strain F3 (b); strain B3 (c); strain B5 (d)

The strain colony morphology is shown in Figure 2. The F1 colony was round, thin, and flat. The mycelium was white during the early stage and green during the middle stage, gradually turning yellow as time progressed. Microscopic observations showed developed mycelium and radially spherical spore heads. The F3 colonies were circular with a convex central portion that was dark green in the middle. The edges of the mycelium were white and tightly distributed, displaying conidial, radially shaped heads.

The B3 colony was light yellow, flat, moist on the surface, jagged, and sticky. The Gram staining result was positive (G+), and the bacteria could be categorized as cocci after microscopic observation of their shape. The B5 colony was white, the distribution of projections, opaque, jagged edges, sticky. The Gram stain was negative (G‐), which was short rod‐shaped under microscope.

**FIGURE 3:**
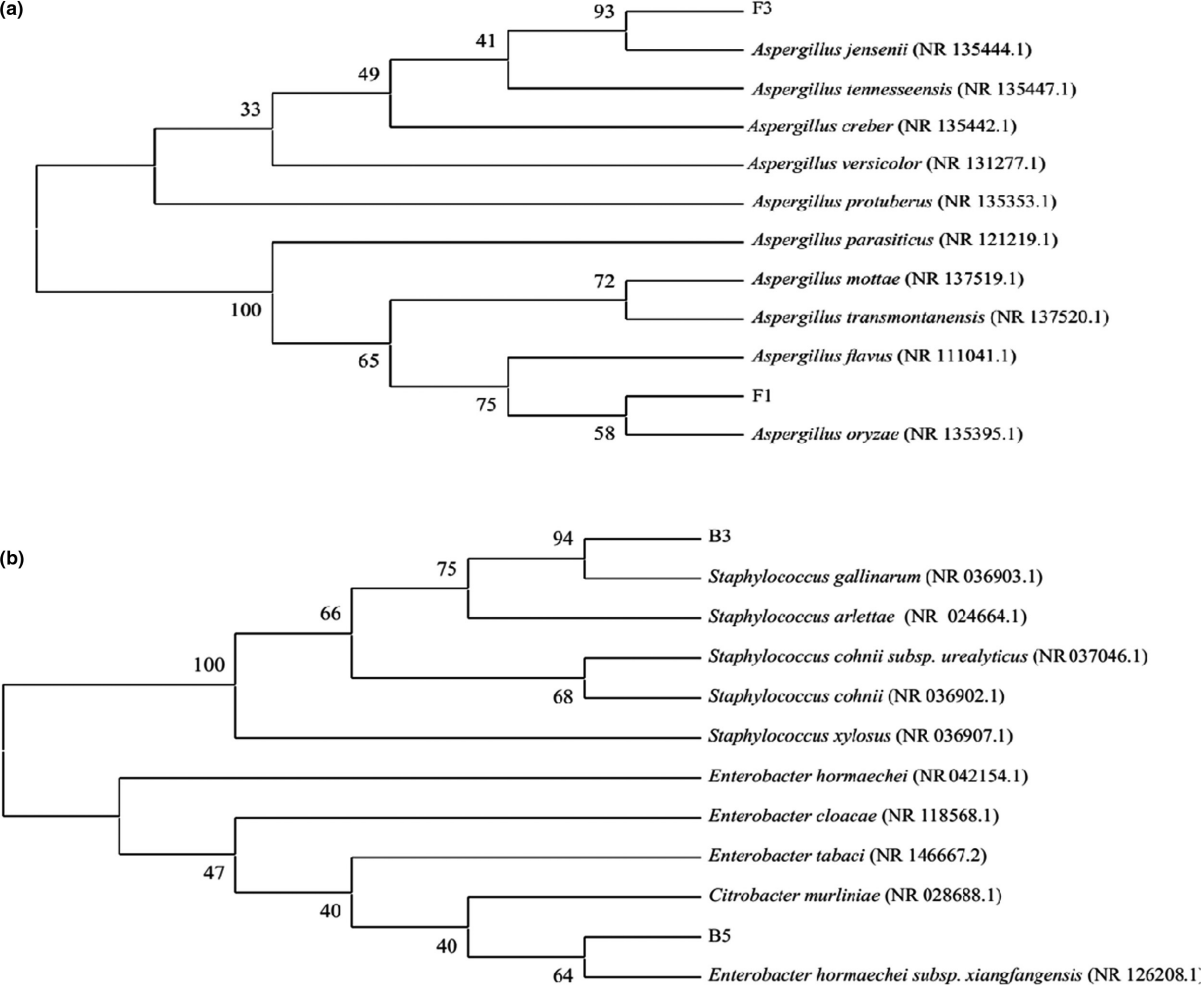
The phylogenetic tree described the correlation between different strains: Phylogenetic tree of F1 and F3 (a); Phylogenetic tree of B3 and B5 (b)

The sequences of the four strains were submitted to the NCBI‐BLAST for comparison, and a phylogenetic tree was constructed using MEGA7.0. The genetic relationship between F1 and *Aspergillus oryzae* appeared on the same branch, while F3 displayed the closest genetic relationship with *Aspergillus jensenii*. Furthermore, B3 and B5 appeared on the same branch as *Staphylococcus gallinarum* and *Enterobacter hormaeche*, respectively, exhibiting the closest relationship (Figure 3). *A. oryzae* is recognized as safe and used in fermented condiments, such as Chinese and Japanese soy sauce, for thousands of years (Ao et al., 2018; Kim, Lim, et al., 2017; Zhao et al., 2019), which can secrete a large amount of protease and carbohydrate hydrolase to decompose the raw materials into short peptides, amino acids, oligosaccharides, and other flavor substances. Studies have shown that the secretion of more *A. oryzae* enzymes is regarded as a basal factor affecting the quality of fermented soybean paste (Yoshino et al., 2011). Additionally, the Koji stage is typically inoculated with *A. oryzae* Huniang 3.042 (Zeljko et al., 2012). *A. jensenii*, which mostly exists in the air and humid environments (Germain et al., 2021), has been revealed as the dominant strain in grain workshop dust (Despot et al., 2016).

*S. gallinarum* is abundant in various fermented products such as Japanese bean paste, in which the microbial community composition has been revealed using polymerase chain reaction‐denaturing gradient gel electrophoresis (Kim et al., 2010). The proteolytic capacity and antibiotic activity of *S. gallinarum* would seem to play an important role in the fermentation of miso (Drosinos et al., 2007; Kempf et al., 2000). Two *Staphylococcus gallisepticum* strains were screened out from the PXDB, revealing a significant inhibitory effect on aflatoxin (Zhang et al., 2020). *Enterobacter hulkeri* has also been detected in many fermented foods, such as fermented bean curd and soybean paste, which could improve flavor formation to a certain extent (Wu et al., 2018).

### 3.2 Strain growth curve and enzymatic characteristics

**FIGURE 4:**
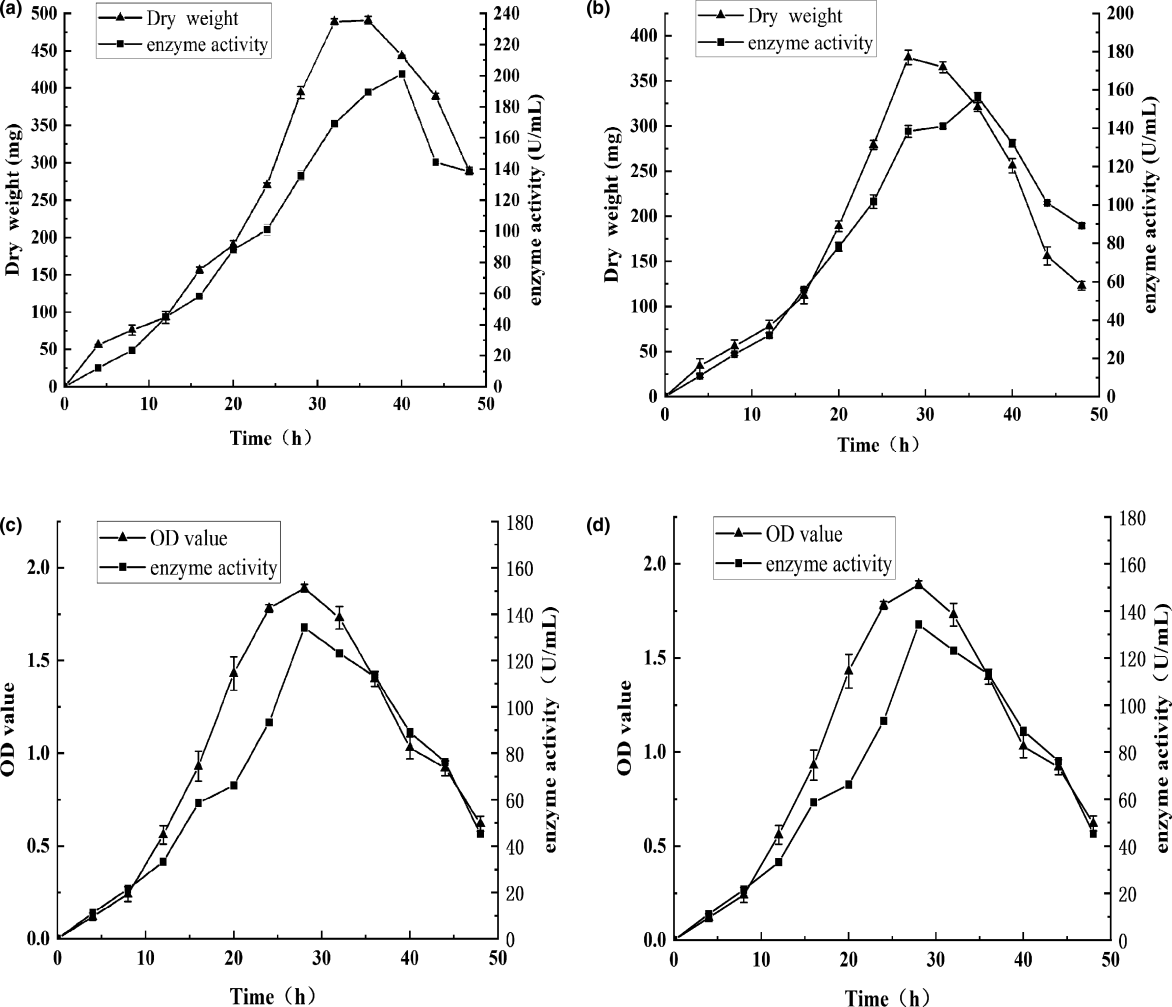
The growth and enzyme production curves of the strains: strain F1 (a); strain F3 (b); strain B3 (c); strain B5 (d). Values are expressed as averages ± standard deviation

The microorganism growth significantly affected enzyme activity. The growth curves of the F1 and F3 strains (Figure 4a,b) indicated that both increased in conjunction with a culture time extension from 0 to 30 h, after which a significant decrease was evident. The enzyme production curve of F1 reached a peak at 40 h, showing enzyme activity of 201.13 ± 1.89 U/ml. F3 displayed a faster growth rate than F1, reaching a peak at 36 h, with enzyme activity of 156.45 ± 2.16 U/ml. Figure 4c,d demonstrate that strains B3 and B5 experience a slow growth period during the first 8 h. B3 entered a stable growth period after 24 h, lasting until 32 h, after which a decline became evident, while the enzyme activity reached a peak at 28 h (134.23 ± 0.42 U/ml). After 20 h, B5 entered a stable growth period, displaying a gradual decline after 28 h, while the enzyme activity reached the highest value of 142.12 ± 0.23 U/ml at 32 h. The enzyme activity initially increased, followed by a decline that was possibly due to nutrient exhaustion, becoming insufficient to sustain strain growth. This led to the formation of large numbers of dormant spores, ultimately reducing the enzyme activity (Liu et al., 2015).

**FIGURE 5:**
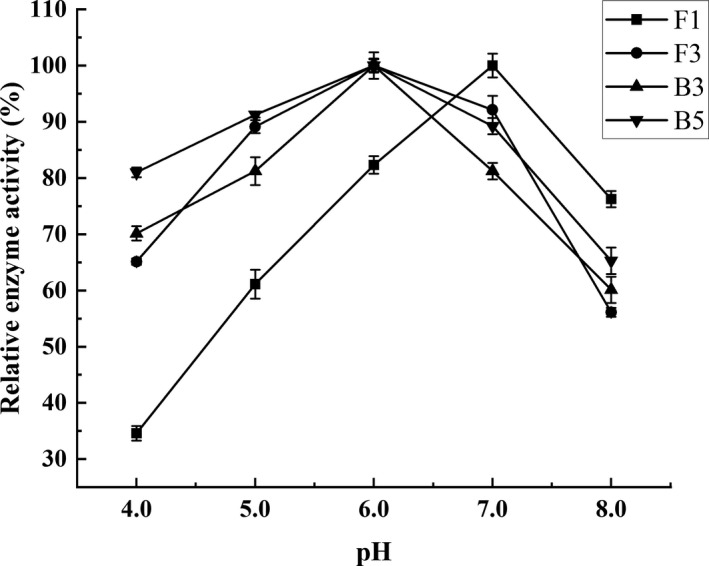
The effect of pH on the protease activity. Values are expressed as averages ± standard deviation

It has been reported that the pH value of PXDB fermentation stage is between 5.0 and 7.0, and the pH values directly affected the enzyme activity (Liu et al., 2015). The protease activity changes of each strain in different pH conditions are presented in Figure 5. Strain F1 displayed significant pH tolerance differences. Under acidic and neutral conditions, the enzyme activity decreased with low pH, and when the pH increased to alkaline conditions, the enzyme activity decreased accordingly. The remaining strains showed a similar tolerance trend to different pH levels, with an optimum pH value of 6.0, indicating that their protease production was suitable for neutral and slightly acidic environments. Furthermore, the protease produced by F3 and B5 was more stable, maintaining an activity level exceeding 80% at a pH value of 5.0.

**FIGURE 6:**
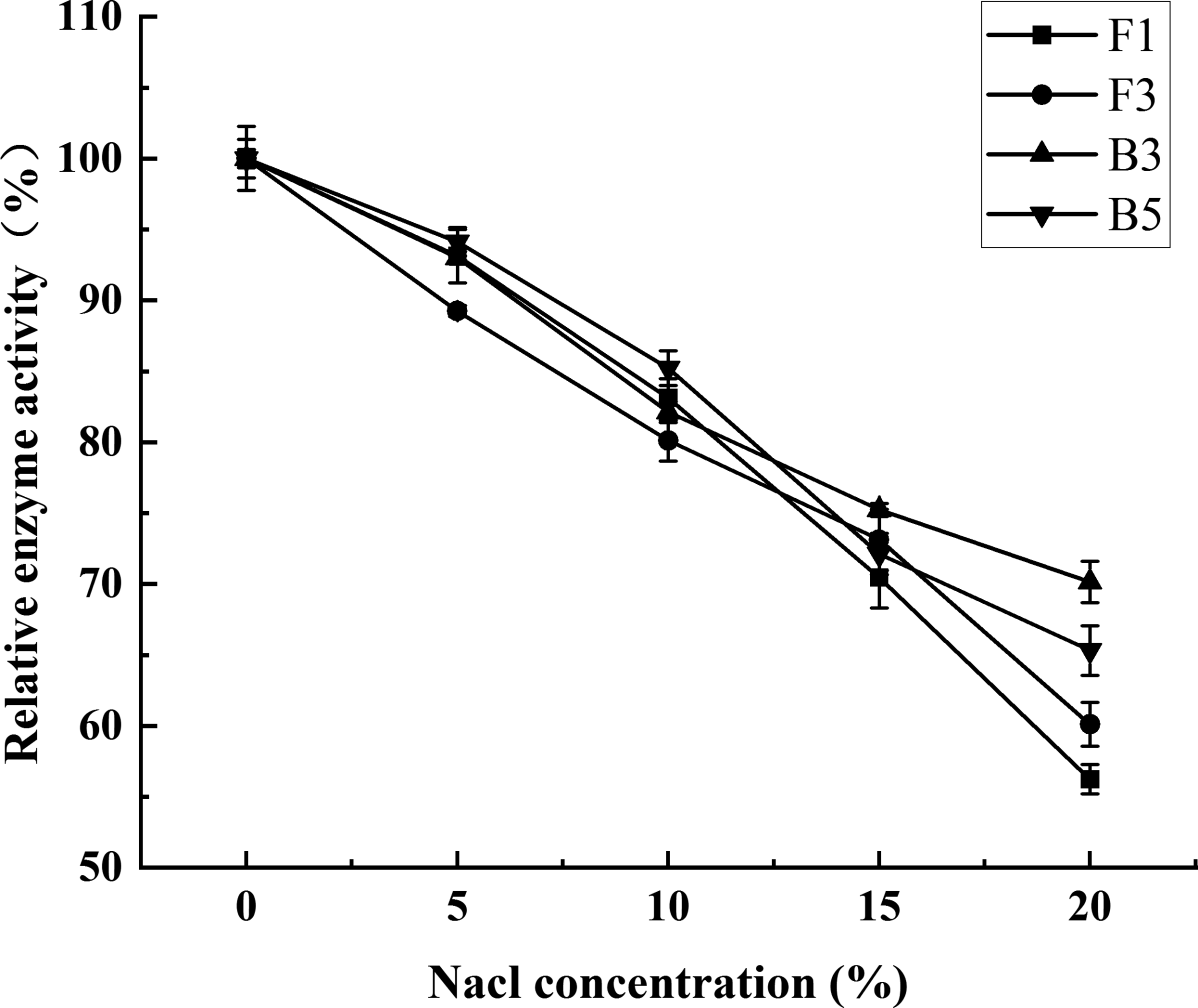
The effect of the NaCl concentration on the protease activity. Values are expressed as averages ± standard deviation

The protease activity was significantly affected by the concentration of NaCl during the Meju fermentation stage. Chun et al. thought that too high or too low salt content would inhibit the activity of protease enzymes in Doenjiang (Chun et al., 2020). An increase in the NaCl concentration resulted in similar protease activity decreased among the various strains (Figure 6). Therefore, the four strains exhibited a certain tolerance to NaCl, maintaining more than 80% of the relative protein activity in 10% NaCl conditions. At a 20% NaCl concentration, F1 displayed the lowest protease activity and B3 the highest.

### 3.3 Analyzing the effect of different broad bean Koji strains on protein degradation

**FIGURE 7:**
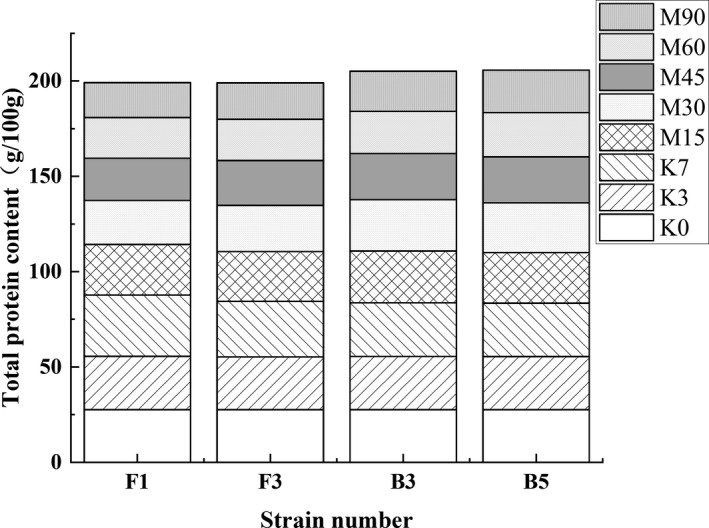
The effect of the different strains on the total protein content during broad bean fermentation

Different strains were inoculated during the broad bean Koji stage of PXDB. The changes in the total protein content are shown in Figure 7. The total protein content of each group increased to a certain extent in conjunction with extended fermentation time due to microbial growth and reproduction. Protein degradation was weak during this alkaline stage (K0‐K7), while the F1 growth and metabolism far exceeded the other strains. During the Meju stage (M15‐M90), the microbes and proteins began to autolyze and degrade, causing a decline in the protein content of each group. Moreover, FG (F1) and FG (F3) decreased rapidly during the first 30 days of the broad bean Meju process, exhibiting a decline in the protein degradation rate after the middle stage. The total protein content was 18.32 ± 0.32 g/100 g and 19.15 ± 0.12 g/100 g, respectively, by the end of the broad bean Meju process.

However, the total protein of FG (B3) and FG (B5) displayed no significant changes during the Koji stage and showed a declining trend in the broad bean Meju. The peptidase activity of FG (B3) and FG (B5) was inhibited by the significantly increased salt content during the early broad bean Meju as shown in Figure 6, while the protein was rapidly degraded after 30 days. However, as the fermentation environment is weakly acidic, the ability of production protease of B3 and B5 gradually increased. The total protein content of FG (B3) and the FG (B5) ultimately decreased to 21.05 ± 0.16 g/100 g and 22.29 ± 0.12 g/100 g, respectively. Overall, the ability of each strain to promote protein degradation was different, with strains F1 and F3 displaying the stronger capacity.

**FIGURE 8:**
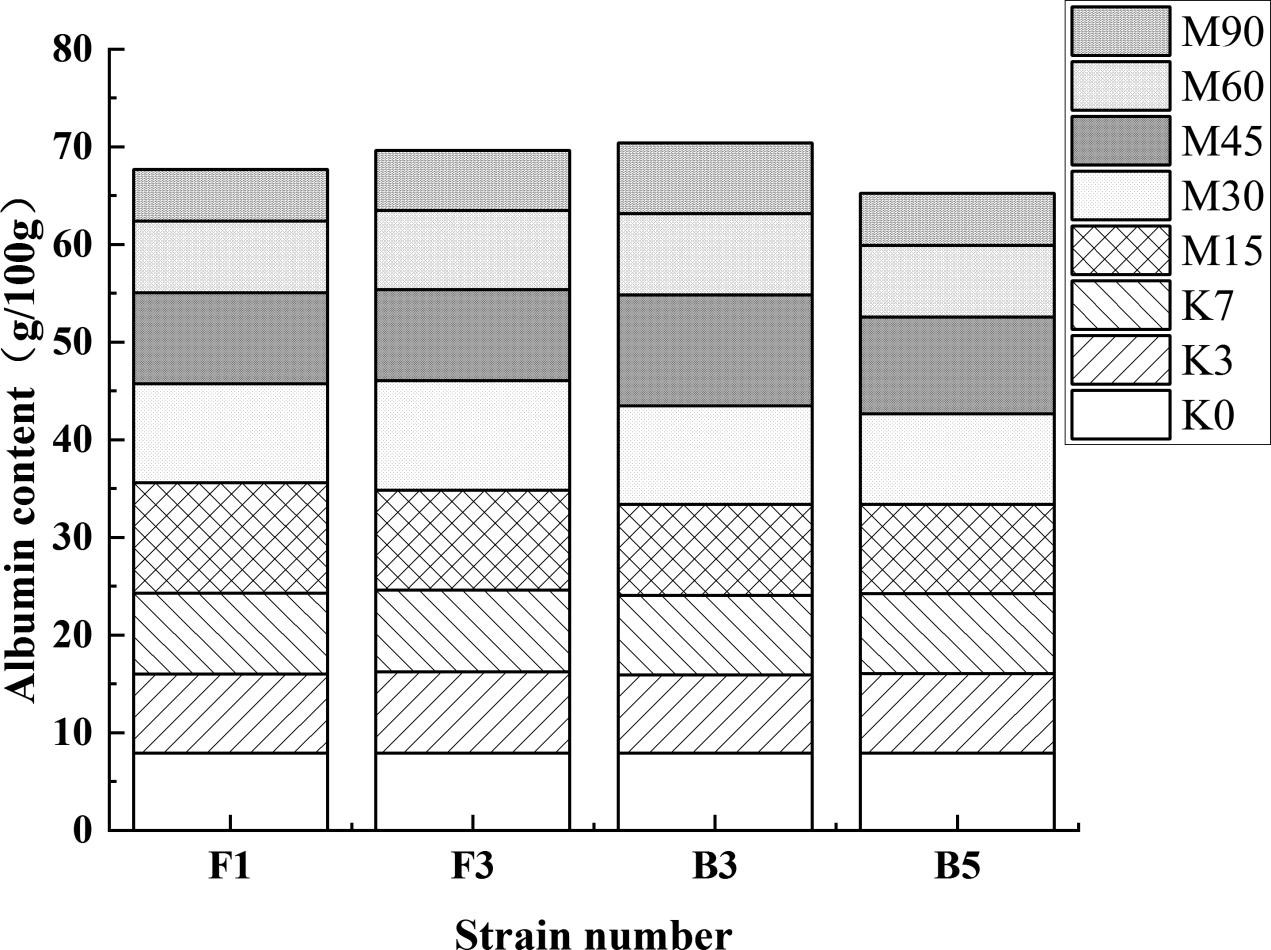
The effect of the different strains on the total albumin content during the broad bean fermentation

Studies have shown that microorganisms produce proteases in fermented food that are primarily responsible for degrading albumin and glutenin (Zhao et al., 2015). As shown in Figure 8, the albumin content of each group remained mostly unchanged in the broad bean Koji. Moreover, the albumin content of FG (F1) and FG (F3) was lower than that of FG (B3) and FG (B5) at the end of the Meju stage, which is 5.32 ± 0.12 g/100 g and 6.15 ± 0.32 g/100 g, respectively. A lower pH level in the fermentation environment may weaken the protein degradation ability, indicating that the four strains promote albumin degradation to a certain extent. However, the time interval for rapid degradation was distinctly different. The protein degradation in FG (F1) and FG (F3) occurred on the 30th day of the Meju stage, while FG (B3) and FG (B5) proteins were degraded on the 45th day. This result could be ascribed to the constantly changing acidic, high‐salt fermentation environment, and the diverse characteristics of the proteases produced by the different strains.

**FIGURE 9:**
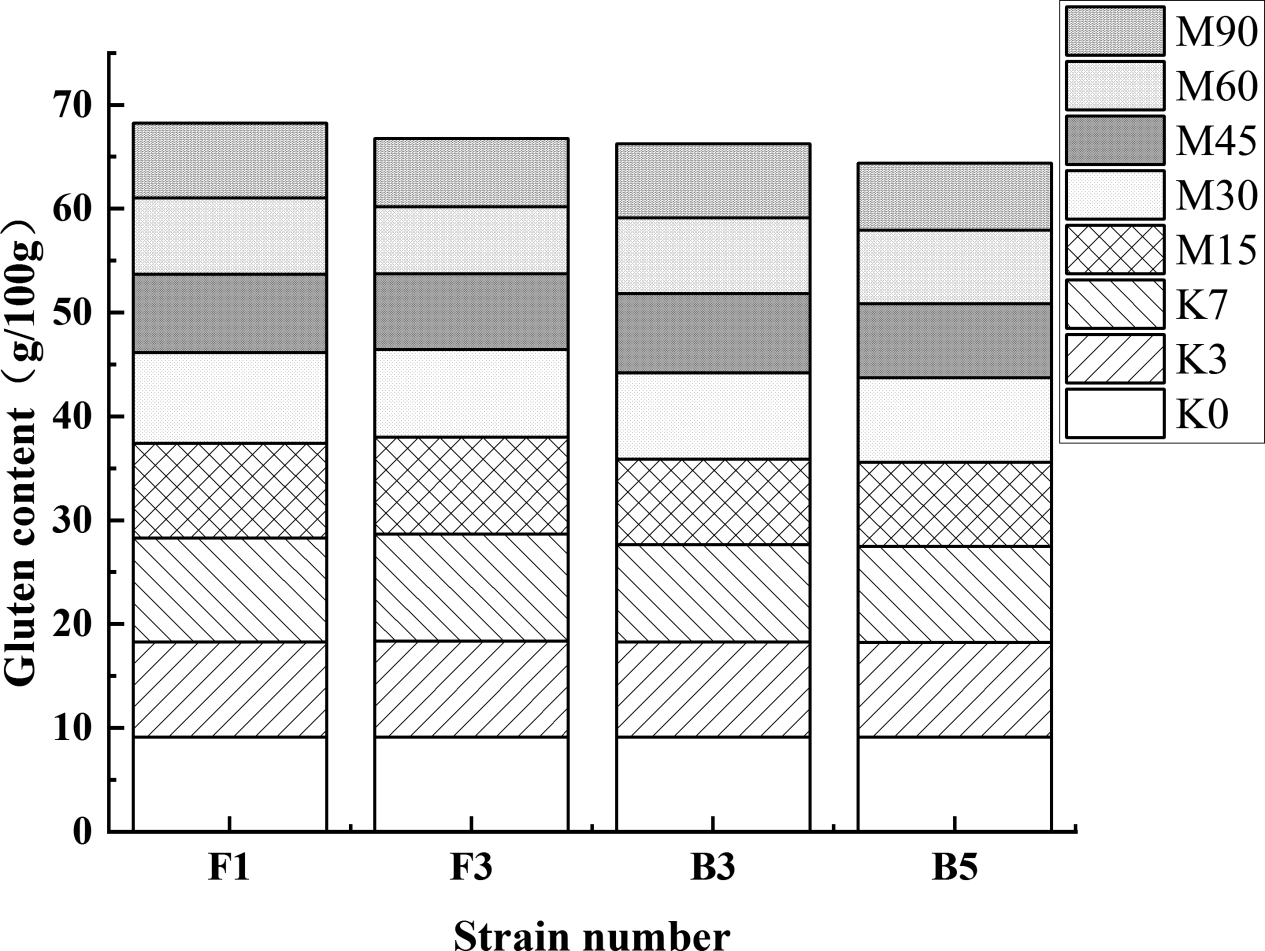
The effect of the different strains on the total glutenin content during the broad bean fermentation

The glutenin content changes in the different fermentation samples are shown in Figure 9. The change in tendencies were similar, with the glutenin content of each group increasing initially, followed by a gradual decrease throughout the fermentation process. The glutenin content of FG (F1) and FG (F3) reached 10.02 ± 0.13 g/100 g and 10.32 ± 0.36 g/100 g on the seventh day of Koji, respectively. Subsequently, the gluten was partially decomposed by the proteases and transformed into small peptides (40). The protease activity decreased during the late Meju fermentation stage due to the constantly changing fermentation environment, weakening protein deterioration, and reducing the overall glutenin degradation rate. At the end of fermentation, the glutenin content of FG (F1), FG (F3), FG (B3), and FG (B5) was 7.21 ± 0.23 g/100 g, 6.56 ± 0.23 g/100 g, 7.13 ± 0.31 g/100 g, and 6.45 ± 0.29 g/100 g, respectively. Each strain exhibited a weaker ability to promote glutenin degradation than albumin, especially F1 and B3. The weak enzymatic tolerance to acidic environments may be responsible for the lower level of glutenin deterioration during the late Meju fermentation stage.

**FIGURE 10:**
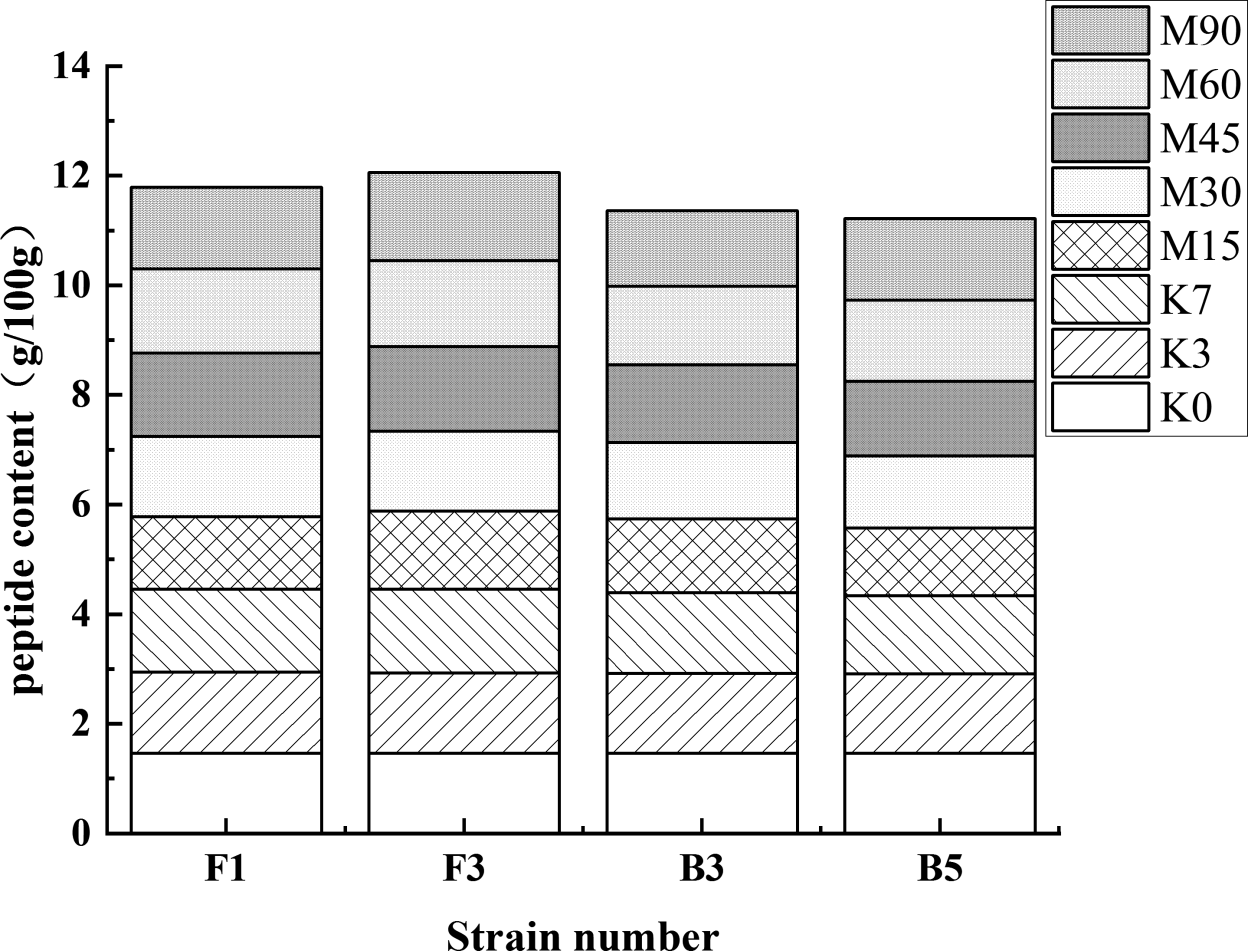
The effect of the different strains on the total polypeptide content during the broad bean fermentation

Peptide content was an important indicator of the degree of protein degradation (Seo & Cho, 2016). The peptide content of each group increased slightly during the Koji stage and decreased significantly during the initial Meju stage (Figure 10). At the end of Meju, the peptide content of FG (F1) and FG (F3) was higher, reaching 11.79 ± 0.04 and 12.06 ± 0.04 mg/g, respectively, followed by FG (B3) and FG (B5) at 11.36 ± 0.04 and 11.22 ± 0.03 mg/g. Therefore, it is speculated that F1 and F3 display stronger protease production ability than B3 and B5. And Zofia et al. believed that protease in the fermentation environment of broad bean would further degrade some peptides into FAAs (Zofiaet al., 2007).

**FIGURE 11:**
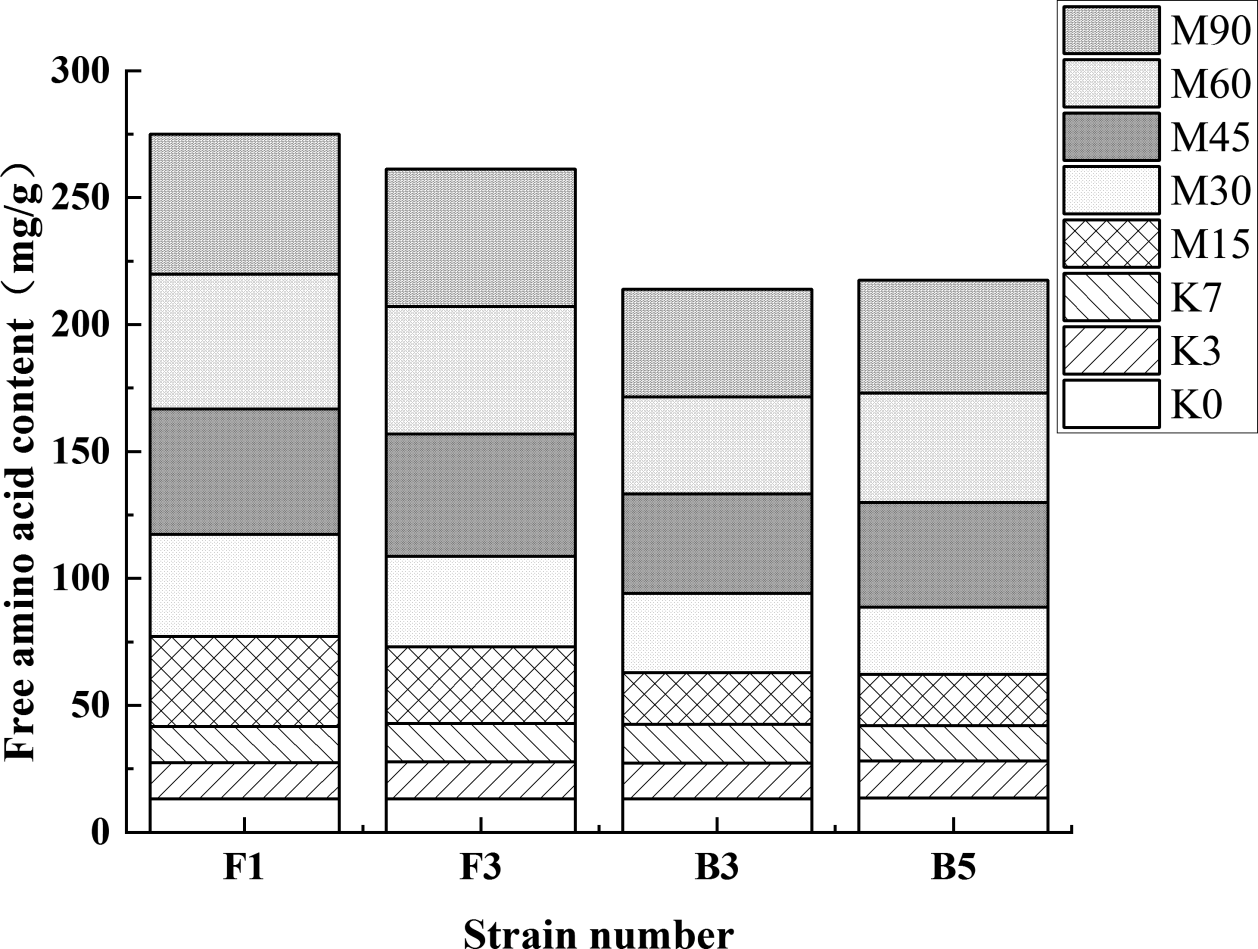
The effects of the different strains on the FAAs content during the broad bean fermentation

The FAAs content changes are shown in Figure 11. Since the Koji stage exhibited microbial growth and enzyme accumulation, the amino acid content of the four sample groups remained mostly unchanged. However, the FAAs content increased rapidly during the early Meju stage. When the broad bean Meju was fermented for 15 days, the FG (F1) amino acid content was the highest, reaching 35.45 ± 2.89 mg/g, followed by FG (F3) at 30.13 ± 2.91 mg/g. The FAAs content displayed an upward trend throughout the fermentation process. Moreover, the FAAs content of FG (F1) and FG (F3) exceeded that of FG (B3) and FG (B5). At the end of fermentation, the amino acid content of FG (F1), FG (F3), FG (B3), and FG (B5) reached 55.12 ± 2.78, 54.11 ± 1.97, 42.56 ± 3.94, and 44.45 ± 2.87 mg/g, respectively. It has been shown that the degree of protein degradation in FG (F1) and FG (F3) was severe, so it may be the cause of the large amount of peptides being decomposed into FAAs. The FAAs in FG (B3) and FG (B5) accumulated rapidly during the mid‐Meju phase. Therefore, it is speculated that B3 and B5 have a good acid protease production ability. The weakly acidic fermentation environment allowed acid protease to act on peptides to produce amino acids.

### 3.4 Analyzing the effect of the different strains on the protein degradation in the simulated system

**FIGURE 12:**
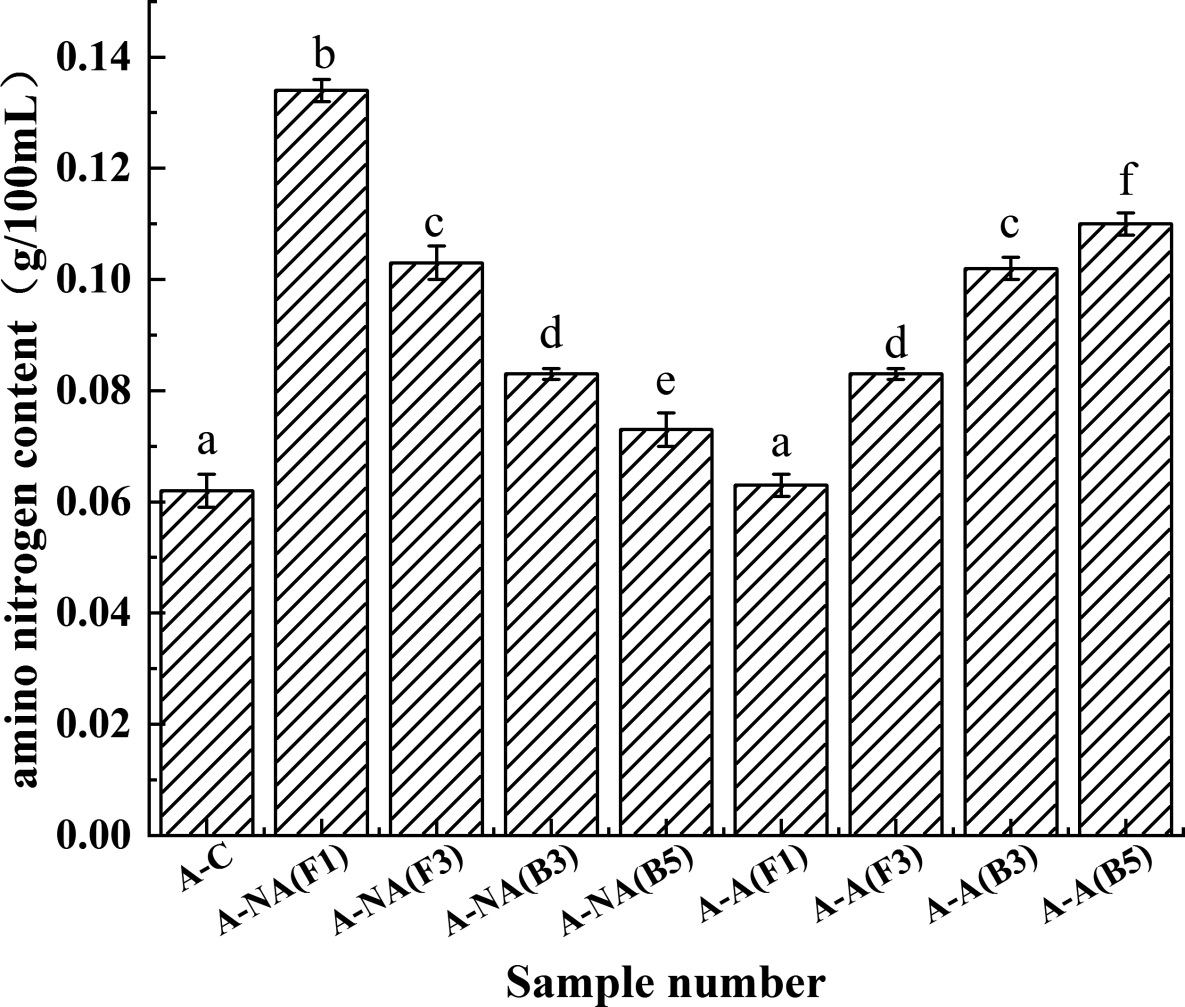
Amino nitrogen changes in the albumin model system. Values are expressed as averages ± standard deviation. Different characters indicate significant differences (*p* < .05) among groups

As a decomposed product of protein, amino acid nitrogen can reflect the degree of protein degradation. The deterioration of albumin and glutenin mainly produced the amino acid nitrogen in the fermented broad beans during the Meju stage. The amino nitrogen changes in the albumin simulation system are shown in Figure 12. In the nonacidified simulation system, A‐NA (F1) displayed the highest amino nitrogen content, reaching 0.134 ± 0.002 g/100 ml, followed by A‐NA (F3) at 0.103 ± 0.003 g/100 ml, while A‐NA (B3) and A‐NA (B5) exhibited a slight increase. These results indicated that the two *Aspergillus* strains demonstrated stronger protease activity in a nonacidified environment and could degrade more albumin to produce peptides and amino acids, as evidenced by the contents of peptide (Figure 10) and FAAs (Figure 11).

However, in the acidified simulation system, the amino nitrogen content in each group showed a contradictory trend. A‐A (B5) displayed the highest amino nitrogen content, reaching 0.110 ± 0.002 g/100 ml, followed by A‐A (B3), and A‐A (F1) amino nitrogen content remains basically unchanged. These results demonstrated that the protease activity of the two bacteria was activated in an acidic environment and began to rapidly decompose the albumin as shown in Figure 8, while the protease produced by the two fungi, especially F1, was inhibited, weakening albumin degradation. And these results were in accordance with the consequence of peptide contents. Therefore, it can be inferred that the protein degradation reaction was primarily concentrated in the early Meju stage of the broad beans fermented with F1 and F3. Furthermore, the bacteria screened from the high salt, and acidic environments began dominating the late fermentation stage, while the impact of the fungal protease on albumin degradation was progressively reduced.

**FIGURE 13:**
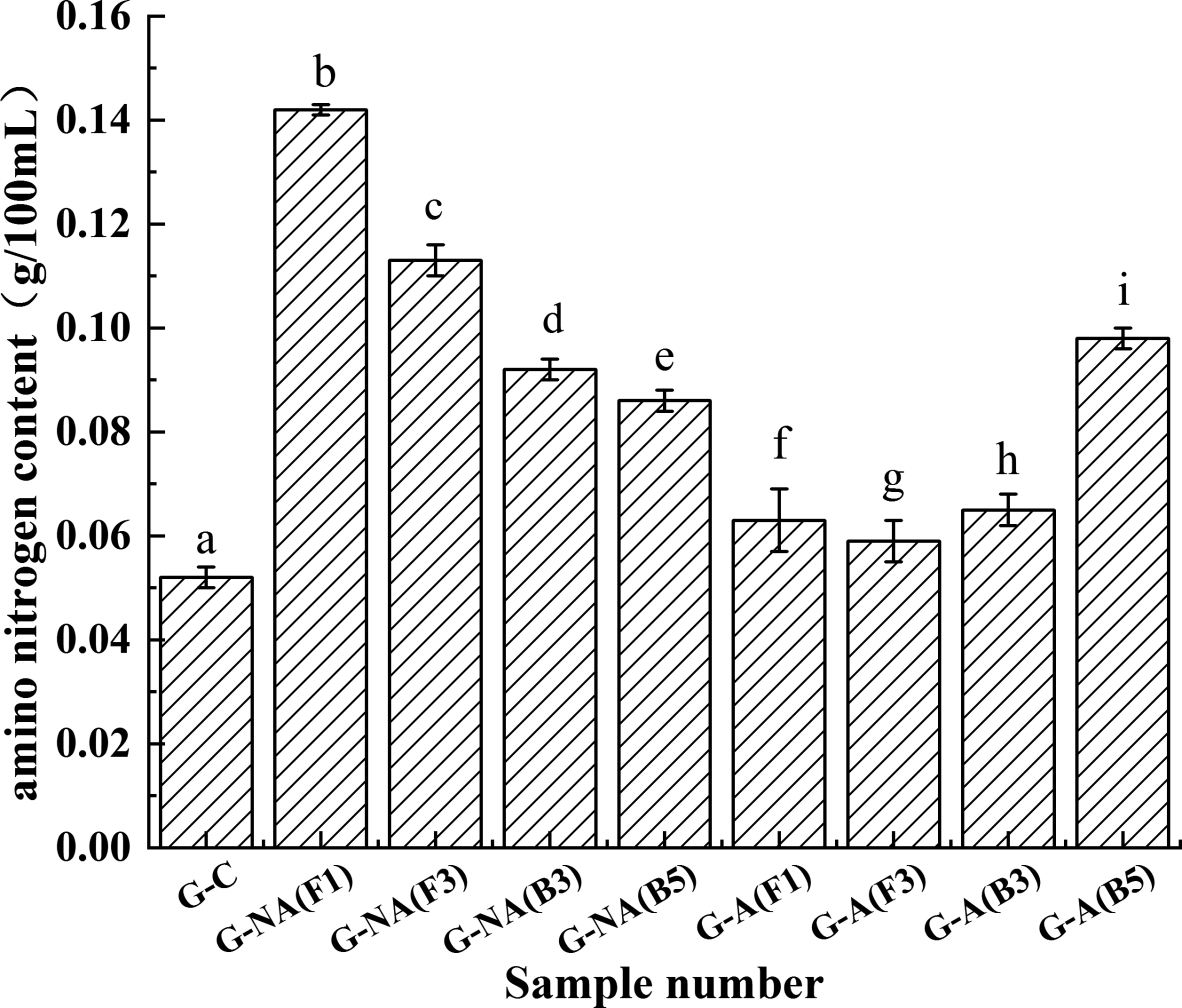
Amino nitrogen changes in the glutenin model system. Values are expressed as averages ± standard deviation. Different characters indicate significant differences (*p* < .05) among groups

In addition, the amino nitrogen content changes in the glutenin simulation system were similar to albumin (Figure 13). G‐NA (F1) displayed the highest amino nitrogen content at 0.142 ± 0.001 g/100 ml, followed by G‐NA (Z3) at 0.113 ± 0.003 g/100 ml, while increased levels were evident in G‐NA (B3) and G‐NA (B5). These findings indicated that the protease produced by the fungi and bacteria could degrade glutenin into polypeptides and amino acids during the early fermentation stage. Of these, the protease derived from fungi plays a dominant role. In the acidified simulation system, the enzyme activity of G‐A (F1), G‐A (F3), and G‐A (B3) was inhibited by the acidic environment, while the enzyme activity of G‐A (B5) showed a higher glutenin degradation ability, with the amino nitrogen content reaching 0.98 ± 0.002 g/100 ml.

Some studies have found that *Aspergillus* only dominated during the early Koji and Meju stages (Zhao, Xu, et al., 2017). In addition, previous research indicated that the protein derived from bacteria was three times that of fungal protein, confirming that bacteria gradually replaced fungi to become dominant during the fermentation process (Wu et al., 2017). Overall, these results of amino nitrogen content proved that bacteria could better adapt to the acidic environment during the late fermentation stage than fungi, while bacterial protease production played a substantial role in the protein degradation reaction.

**FIGURE 14:**
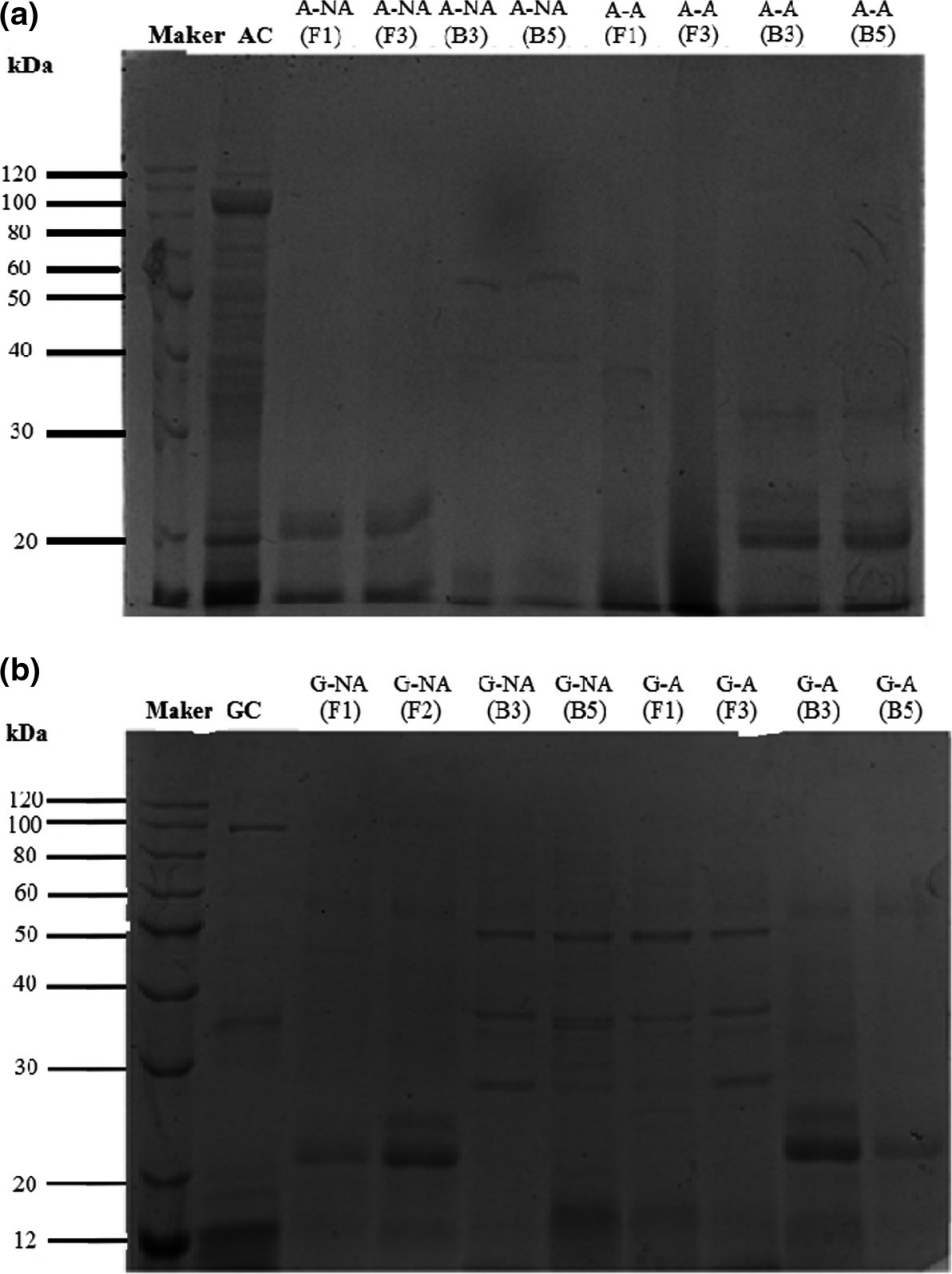
The sodium dodecyl sulfate‐polyacrylamide gel electrophoresis profiles of the proteins in the model system: The sodium dodecyl sulfate‐polyacrylamide gel electrophoresis profiles of the albumin protein in the model system (a); The sodium dodecyl sulfate‐polyacrylamide gel electrophoresis profiles of the glutenin protein in the model system (b)

SDS‐PAGE was used to analyze the protein degradation in the different systems. In the nonacidified system, the experimental group displayed distinct changes compared with the control group, indicating various degrees of albumin deterioration in each group (Figure 14a). A‐NA (F1) and A‐NA (F3) displayed a clear band at 20 kDa, indicating a higher degree of albumin decomposition into polypeptides. A‐NA (B3) and A‐NA (B5) displayed bands at 60 and 40 kDa, respectively, while the low‐molecular‐weight bands disappeared Moreover, in the acidified simulation system, the two bands of the A‐A (F1) group were located at large molecular weights of 50 and 40 kDa, owing to the inhibition of their enzyme activity reducing protein degradation and small peptide chain production. Notably, the A‐A (B3) and A‐A (B5) bands were mainly concentrated below 30 kDa, indicating the activation of the protease produced by the acidified simulation system, while the large albumins were mostly degraded into small peptides.

The electrophoresis diagram of the glutenin simulation system is shown in Figure 14b. In the nonacidified simulation system, the G‐NA (F1) bands were all concentrated around 20 kDa, followed by G‐NA (F3) with two distinct bands between 20 and 30 kDa, indicating that most of the gluten was degraded into small molecular subunits. The results showed that the protease produced by F1 and F3 significantly affected glutenin degradation during the early Meju stage. The G‐A (F1) and G‐A (F3) bands were distributed between 20 and 50 kDa in the acidified simulation system. Compared with the nonacidified simulation conditions, the fungal protease was significantly inhibited, while the glutenin degradation ability was reduced. The bands of G‐A (B3) and G‐A (B5) were distributed between 20 and 30 kDa.

Combined with the amino nitrogen content changes (Figure 13), the protease produced by B5 displayed a significant impact on glutenin degradation in the acidified simulation system. Overall, these results indicated that the F1 and F3 protease was inhibited as the pH decreased, while that of B3 and B5 showed a stronger ability to degrade glutenin. Considering the conditions in the two simulation systems, it is speculated that the protease produced by the fungi promoted protein degradation during the early Meju stage. During the later stage of fermentation, the proteases produced by the bacteria significantly affected protein deterioration with the accumulation of acidic substances in the environment and a decrease in the pH value.

## 4 CONCLUSION

Overall, the findings showed that the four strains were *Aspergillus oryzae*, *Aspergillus jensenii*, *Staphylococcus gallinarum*, *Enterobacter hormaeche*, and *Aspergillus oryzae*. Furthermore, the total protein, peptides, and amino acids of the fungus experimental groups (F) were higher than the bacterial experimental groups (B). In addition, the enzyme system produced by fungi exhibited a stronger ability for albumin (20 kDa) and glutenin (<30 kDa) deterioration in neutral conditions, while the bacterial enzyme system was more efficient in degrading albumin (<30 kDa) and glutenin (20–30 kDa) in acidic conditions, as indicated by SDS‐PAGE. In conclusion, the research found that bacteria and fungi played an important role in the degradation of protein during different fermentation stages of broad bean fermentation. Together, they participated in the degradation of albumin and glutenin to form peptides and amino acids, which provided a material basis for the formation of PXDB flavor substances.

## CONFLICT OF INTEREST

The authors declare that there are no conflicts of interest regarding the publication of this paper.

## Data Availability

The data used to support the findings of this study have not been made available because [REASON].
